# The protective effect of topical rifamycin treatment against sternal wound infection in diabetic patients undergoing on-pump coronary artery bypass graft surgery

**DOI:** 10.5830/CVJA-2014-008

**Published:** 2014-06

**Authors:** Fatih Aygun, Ahmet Kuzgun, Seref Ulucan, Ahmet Keser, Mahmut Akpek, Mehmet G Kaya

**Affiliations:** Department of Cardiovascular Surgery, School of Medicine, Mevlana University, Konya, Turkey; Department of Cardiovascular Surgery, School of Medicine, Mevlana University, Konya, Turkey; Department of Cardiology, School of Medicine, Mevlana University, Konya, Turkey; Department of Cardiology, School of Medicine, Mevlana University, Konya, Turkey; Department of Cardiology, School of Medicine, Erciyes University, Kayseri, Turkey; Department of Cardiology, School of Medicine, Erciyes University, Kayseri, Turkey

**Keywords:** rifamycin, sternal wound infection, on-pump CABG

## Abstract

**Objectives:**

The aim of this study was to investigate the protective effect of topical rifamycin SV treatment against sternal wound infection (SWI) in diabetic patients undergoing on-pump coronary artery bypass graft (CABG) surgery.

**Methods:**

One hundred and fifty-nine diabetic patients who were scheduled to undergo isolated on-pump CABG surgery were included. Eight were excluded for various reasons. Of the 151 patients, 51 were on insulin therapy and 100 were on oral anti-diabetics. The risk of mediastinitis was assessed using the American College of Cardiology/American Heart Association 2004 guideline update for CABG surgery. According to the risk scores, patients were divided into two comparable groups: the rifamycin group (*n* = 78) received topical rifamycin treatment after on-pump CABG surgery, and the control group (*n* = 73) received no topical treatment.

**Results:**

Deep sternal wound infection (mediastinitis) was not observed in either group (0/78 vs 0/73, *p* = 1.0). No superficial sternal wound infection was observed in the rifamycin group, however, it did occur in one patient in the control group (0/78 vs 1/73, *p* = 0.303). Wound culture was performed and coagulase-negative staphylococci were observed. The infection regressed on initiation of antibiotic therapy against isolated bacteria and the patient was discharged after a full recovery.

**Conclusion:**

Although the difference in rate of superficial sternal wound infection (SSWI) in the rifamycin and control groups was not statistically significant, locally applied rifamycin SV during closure of the sternum in the CABG operation may have had a protective affect against SWI.

## Abstract

Sternal wound infection (SWI) is a rare complication occurring after coronary artery bypass graft (CABG) surgery. Sternal wound infection occurs in one to 3% of patients and has a mortality rate of up to 40%. It is also associated with prolonged hospital stay and increased healthcare costs.^1^-^4^

According to the American College of Cardiology/American Heart Association (ACC/AHA) 2004 guideline update for CABG surgery, the risk of mediastinitis is evaluated before CABG surgery using factors, such as age of patient, the presence of obesity, diabetes or chronic obstructive pulmonary disease (COPD), the need for dialysis, an ejection fraction (EF) < 40%, and being scheduled for emergency surgery.^5^

In studies by Khanlari *et al.* and Kloos *et al.*, patients with SWI were divided into two subgroups: superficial sternal wound infection (SSWI) and deep sternal wound infection (DSWI).^6^,^7^ While SSWI involves only subcutaneous tissue, DSWI is associated with sternal osteomyelitis and sometimes with infected retrosternal space (termed mediastinitis). These researchers reported that DSWI occurred in 0.25 to 2.3% of patients.^6^,^7^

Rifamycin SV is a relatively effective agent for the treatment of gram-positive bacteria, *Mycobacterium tuberculosis* and certain gram-negative bacteria. Rifampicin, derived from rifamycin SV, is readily absorbed after oral administration and possesses higher antimicrobial activity against *Staphylococcus aureus S epidermidis, Streptococcus viridans*, and *Mycobacterium tuberculosis*, even in very low doses. In only one study in the literature has the use of antibiotics containing rifampicin been suggested to improve outcomes in staphylococcal deep-wound infections.^6^

In the present study, we aimed to investigate the protective effects of topical rifamycin SV treatment on SWI after on-pump CABG surgery in diabetic patients.

## Methods

One hundred and fifty-nine diabetic patients who were scheduled to undergo isolated CABG surgery in the Department of Cardiovascular Surgery, Mevlana University between July 2008 and July 2011 were prospectively enrolled. Of these patients, eight were excluded due to use of the intra-operative beating-heart technique, a need for revision in the post-operative period, or death.

In the remaining 151 patients, the risk of mediastinitis was assessed according to the ACC/AHA 2004 guideline update for CABG surgery.^5^ We grouped the patients according to their mediastinitis risk scores into two comparable groups: the rifamycin group consisted of 78 patients (52 male, mean age 62 ± 8 years) who received local antibiotic rifamycin SV i.m. (Rif® 250 mg/3-ml ampoule) on the sternal region after CABG surgery, and the control group consisted of 73 patients (45 male, mean age 61 ± 8 years). They did not receive a local antibiotic.

The local ethics committee approved the study. Written informed consent was obtained from the patients. It was determined prior to the initiation of the study that patients developing SSWI would be treated by the administration of antibiotics alone. Patients developing DSWI would be treated by the administration of antibiotics plus surgery.

During the pre-operative period, all patients were assessed for the risk of mediastinitis according to the ACC/AHA 2004 guideline for CABG surgery,^5^ using eight parameters including age, presence of obesity, diabetes or COPD, the need for dialysis, ejection fraction (EF) < 40%, and scheduled for emergency surgery. Baseline characteristics, parameters used to assess the risk of mediastinitis, and post- and intra-operative data of the patients are presented in Table 1.

**Table 1:** Baseline clinical characteristics of the study groups.

	*Group 1 (n = 78)*	*Group 2 (n = 73)*	p*-value*
Age (years)	62 ± 8	61 ± 8	0.605
Sex (F/M)	26/52	28/45	0.635
BMI (kg/m^2^)	28.9 ± 4.6	29.1 ± 4.2	0.796
Mediastinitis risk score	0.7 ± 0.4	0.7 ± 0.4	0.570
Number of grafts (*n*)	3.2 ± 1.0	3.3 ± 1.0	0.557
CABG time (min)	104 ± 30	105 ± 27	0.896
Cross-clamp (min)	70 ± 21	71 ± 20	0.687
24-hour drainage (ml)	508 ± 200	549 ± 317	0.350
Total drainage (ml)	515 ± 202	587 ± 334	0.113
COPD	6 (7.7%)	4 (5.5%)	0.746
Dialysis	2 (2.6%)	3 (4.1%)	0.673
Ejection fraction (< 40%)	13 (16.7%)	12 (16.4%)	0.856
Urgent surgery	1 (1.3%)	3 (4.1%)	0.353
Emergency surgery	0 (0%)	0 (0%)	1.0
Sternal infection	0 (0%)	1 (1.4%)	0.303

Categorical variables are expressed as number (percentage) and continuous variables as mean = SD. BMI = body mass index; CABG = coronary artery bypass graft; COPD = chronic obstructive pulmonary disease.

Skin cleansing was performed in all patients prior to surgery. Combined insulin therapy with regular human insulin (Humulin® R 100 U/ml) and insulin glargine (Lantus® 100 U/ml) was administered to control blood glucose levels below 200 mg/dl during pre-, intra- and postoperative periods. Insulin infusion was initiated in patients as required. The standard prophylactic antibiotic regimen used in our clinic was administered to patients, that is 1 g cefazolin sodium (Cefamezin-IM/IV®) 30 minutes before surgery and 1 g every eight hours after surgery for 48 hours.

Cardiopulmonary bypass (CPB) duration, cross-clamping times and number of grafts in both groups are shown in Table 1. Only left internal mammary artery grafts were used in all patients. Meticulous aseptic techniques were used during the operation and unnecessary use of electrocautery and excessive perfusion in CPB were avoided.

All patients were kept in the intensive care unit for 24 hours and the patients were referred to a regular ward within the second 24 hours after drains and arterial catheters were removed. Central venous catheters were removed on the second postoperative day. The patients were discharged on postoperative day 6 ± 3.

In the rifamycin group, mediastinum, sternum and suprasternal tissues were irrigated after surgery using rifamycin SV i.m. (Rif® 250 mg/3-ml ampoule) diluted with 10 ml isotonic solution. In the control group, irrigation was not performed. The two groups were compared with regard to risk for sternal infection.

## Statistical analysis

Statistical analysis was performed using statistical package for social sciences 13.0 (SPSS Inc, Chicago, IL, USA). The Kolmogorov-Smirnov test was used to determine the distribution of numerical parameters. Continuous variables are presented as mean ± standard deviation. For comparison of independent continuous variables, the Student’s *t*-test or Mann–Whitney *U*-test was used where appropriate. Categorical data were compared using the Fisher’s exact test or chi-square test. For all statistics, a *p*-value < 0.05 was considered statistically significant.

## Results

There were no significant differences between the two groups in terms of baseline characteristics and mediastinitis risk percentages (Table 1).

The patients were followed up for the development of SWI for 30 days after the surgery. In neither group did DSWI occur. While no SSWI was observed in the rifamycin group, it was observed in one patient in the control group (0/78 vs 1/73, *p* = 0.303). This patient, who used oral anti-diabetic medication, was 75 years old and had a serum creatinine level below 2.5 mg/dl, had a low risk profile (total risk score: 3 and pre-operative mediastinitis risk percentage: 0.5%), according to the ACC/AHA 2004 guideline.^5^

Wound culture was performed and coagulase-negative staphylococci (CoNS) were observed. The patient was put on appropriate antibiotic therapy with sodium fusidate (Stafine® tablet 500 mg) three times daily and rifampicin (Rifcap® capsule 150 mg) twice daily. The infection regressed and the patient was discharged after a full recovery.

The amount of drainage in the control group, particularly in four patients, was higher than in the patients in the rifamycin group, however, the difference was not statistically significant. This was attributed to the pre-operatively administered antiplatelet agents rather than to surgical reasons, and re-exploration was not required. However, none of the four patients developed sternal infection. None of the patients required re-exploration due to bleeding, tamponade or for other reasons.

## Discussion

Rifamycin was first isolated in 1957 from a fermentation culture of Nocardia mediterranei and used as a novel antibiotic compound. Rifamycin SV is a relatively effective agent for the treatment of gram-positive bacteria, Mycobacterium tuberculosis and certain gram-negative bacteria. Rifampicin, an orally active agent that possesses higher antimicrobial activity, is derived from rifamycin SV. It has lower antimicrobial activity compared to its orally active derivative of rifampicin; however, both are effective against gram-positive cocci, especially staphylococci. Moreover, they possess higher antimicrobial activity against Staphylococcus aureus, S epidermidis, Streptococcus viridans and Mycobacterium tuberculosis, even in very low doses. There is only one study reporting improved outcomes in DSWI with the use of rifampicin.^6^

CoNS are part of normal skin flora.^7^ They are omnipresent and cause infection in patients as well as in hospital staff.^7^,^8^ CoNS are multiple-drug-resistant pathogens that can infect deep surgical wounds and have the potential to threaten life.^9^ Stahle *et al.*^10^ reported the rate of CoNS in surgical wound infections as 14%. It is known that CoNS are also the predominant bacteria in DSWI.^11^

SWI are divided into two subgroups: superficial sternal wound infection (SSWI) and deep sternal wound infection (DSWI). While SSWI involves only subcutaneous tissue, DSWI is associated with sternal osteomyelitis and sometimes with infected retrosternal space (termed mediastinitis).^12^ Studies have reported that DSWI occurs in 0.25 to 2.3% of patients.^13^-^17^

While re-opening and debridement of the mediastinum is required in the treatment of DSWI, administration of antibiotics is generally sufficient to treat SSWI. In the present study, only one patient (1/151, 0.66%) in the control group developed SSWI and was treated with the administration of antibiotics.

DSWI occurring after CABG operation has a multifactorial aetiology, with a potential risk of death and high hospital costs.^18^ Many studies have suggested the underlying aetiology of DSWI occurring after CABG to be obesity, advanced age, prolonged CPB duration, diabetes, high creatinine levels, use of bilateral internal mammary artery grafts, and unnecessary use of electrocautery.^14^,^18^-^21^ Recent studies have suggested that DSWI is associated with obesity and re-operation, and also indicated that use of bilateral internal mammary artery grafts, duration and complexity of the operation, and diabetes are other risk factors.^22^

It is well known that mobilisation of the internal mammary artery causes sternal devascularisation and the resultant ischaemia contributes to sternal dehiscence or infection.^14^,^22^ In the present study, according to the ACC/AHA 2004 guideline,^5^ the pre-operative mediastinitis risk percentage of one patient who developed SSWI was 0.5%, due to the risk factors, advanced age and the presence of diabetes. Although this patient was not a dialysis patient, he/she had a high creatinine level (2.5 mg/dl).

In a 10-year retrospective study of 5 440 patients who underwent cardiac surgery, Khanlari *et al.*^6^ evaluated 100 patients with staphylococcal DSWI developing after cardiac surgery. They reported that a rifampicin-containing antibiotic regimen significantly improved the outcomes during a one-year follow-up period.

Many factors have been implicated in the occurrence of DSWI after cardiac surgery. However, there is no consensus on which is the most important and best predictive factor.^23^ On the other hand, diabetes has emerged as a significant risk factor of cardiovascular surgeons, for the development of DSWI after CABG operation. In terms of the pathophysiological consequences of diabetes, microvascular changes and elevated blood glucose levels impair the healing process of surgical wounds.^24^,^25^ The present study is distinctive in that it examined patients who were on oral anti-diabetic agents or insulin therapy.

## Conclusion

Although the difference in the rate of superficial sternal wound infection between the rifamycin and control groups was not statistically significant, locally applied rifamycin SV during closure of the sternum after CABG surgery may have had a protective affect against SWI.
